# Age-Specific Associations Between eHealth Literacy and Sleep Quality Among Adults: Cross-Sectional Study

**DOI:** 10.2196/75813

**Published:** 2025-12-24

**Authors:** Yujie Liu, Wenjie Xue, Yuhui Sheng, Suping Wang, Ruijie Gong, Shangbin Liu, Chen Xu, Yong Cai

**Affiliations:** 1 Public Health Research Center, Tongren Hospital, Shanghai Jiao Tong University School of Medicine, 1111 Xianxia Road, Shanghai, 200335, China, 86 021-52039999; 2 The Ninth People’s Hospital, Huangpu Branch, Shanghai Jiao Tong University School of Medicine, Shanghai, China; 3 Clinical School of Medicine, Chengdu University of Traditional Chinese Medicine, Chengdu, China; 4 Shanghai Jiao Tong University School of Medicine, Shanghai, China; 5 Shanghai Xuhui District Centre for Disease Control and Prevention, Shanghai, China; 6 Institute of Community Medicine, China Academy of Hospital Development, Shanghai Jiao Tong University, Shanghai, China

**Keywords:** eHealth literacy, sleep quality, digital health, emerging adults, established adults, middle-aged adults, health disparities

## Abstract

**Background:**

Young and middle-aged adults are vulnerable to poor sleep quality. eHealth literacy, defined as the ability to effectively access and use digital health information, has been linked to improved health behaviors and may promote better sleep outcomes. However, its relationship with sleep quality remains unclear, especially across age groups. Age-related disparities in eHealth literacy may contribute to a digital health divide in sleep outcomes.

**Objective:**

This study aimed to examine the relationship between eHealth literacy and sleep quality among adults aged 18 to 59 years in Shanghai, China, as well as explore age-stratified effects.

**Methods:**

A cross-sectional study was conducted between October and December 2022 in 3 districts of Shanghai, with 7 community health service centers randomly selected. Participants were recruited through convenience sampling to complete an online survey. eHealth literacy was assessed using the eHealth Literacy Scale, and sleep quality was measured using the Pittsburgh Sleep Quality Index. Covariates included sociodemographic characteristics, health status, and health behaviors. Logistic regression models were applied to examine the relationship between eHealth literacy and sleep quality, with stratified analyses conducted by age (emerging adults [18-29 years], established adults [30-45 years], and middle-aged adults [46-59 years]).

**Results:**

A total of 1810 participants completed the survey. The prevalence of poor sleep quality was 37.9% (686/1810). Participants with eHealth literacy scores in the 25th to 75th percentile range (odds ratio [OR] 1.594, 95% CI 1.216-2.089, *P*<.001) and below the 25th percentile (OR 1.584, 95% CI 1.149-2.182, *P*=.005) had a significantly higher likelihood of reporting poor sleep quality compared to those with scores above the 75th percentile. Age-stratified analysis indicated that this association was significant only among emerging adults (OR 2.491, 95% CI 1.133-5.479, *P*=.02 for scores between the 25th and 75th percentiles; OR 2.975, 95% CI 1.230-7.195, *P*=.02 for scores below the 25th percentile) and established adults (OR 1.439, 95% CI 1.001-2.067, *P*=.049 for scores between the 25th and 75th percentiles).

**Conclusions:**

This study found that eHealth literacy was associated with sleep quality among younger participants but not middle-aged ones, highlighting the digital divide in sleep health. These findings suggest that enhancing eHealth literacy may serve as an effective strategy for improving sleep outcomes. However, to ensure equitable health outcomes, interventions should be tailored to address the age-specific needs and varying levels of digital access across different groups.

## Introduction

Sleep is a fundamental physiological process essential for maintaining physical health, mental well-being, and overall quality of life. However, it remains an underrecognized priority in public health agendas, particularly in low- and middle-income countries [1]. According to the 2025 China National Health Sleep White Paper, sleep quality among residents remains suboptimal, with approximately 64% experiencing sleep disturbances once or twice per week. [2]. The prevalence and severity of sleep disturbances vary across age groups. Young and middle-aged adults, as a core segment of the workforce, are particularly vulnerable to sleep disturbances due to occupational stress, long working hours, and irregular schedules [3-5]. Chronic sleep disturbance impairs stress management and exacerbates emotional distress while also being associated with poorer health outcomes, placing a burden on health care systems and reducing workplace productivity [6,7].

Sleep quality is influenced by multiple factors, including socioeconomic, physiological, psychological, and behavioral elements [8,9]. While these factors contribute to variations in sleep quality, increasing attention has been directed toward the role of eHealth literacy in health management. Defined as an individual’s ability to access, understand, evaluate, and apply health information from digital sources to make informed health decisions [10], eHealth literacy has been shown to facilitate changes in health-related behaviors by bridging the gap between health information acquisition and actionable practices [11]. A systematic review further confirmed that eHealth literacy is associated with positive outcomes, including improved health behaviors, better psychological well-being, and increased use of health services [12]. These findings suggest that individuals with higher eHealth literacy are better equipped to adopt and maintain health-promoting behaviors that improve sleep outcomes.

While the significance of eHealth literacy in facilitating health-related behavior changes is recognized, its specific impact on sleep quality remains inadequately investigated. Some studies have suggested potential pathways through which eHealth literacy may influence sleep quality. For example, higher eHealth literacy has been associated with greater adherence to sleep hygiene practices [13], potentially by enhancing individuals’ ability to identify and apply credible health information, thereby promoting better sleep quality. Another study indicated that higher eHealth literacy could reduce the risk of cyberchondria, which is subsequently associated with improved sleep quality [14]. However, evidence regarding the direct relationship between eHealth literacy and sleep quality is limited.

The association between eHealth literacy and sleep quality may vary by age. Previous research indicates that eHealth literacy is typically higher among younger populations [15,16]. Younger adults tend to engage more with digital health resources and may benefit significantly from them in managing sleep-related issues [17]. In contrast, middle-aged adults often face barriers in accessing and using such tools effectively despite a growing need for sleep management as sleep quality tends to decline with age [18,19]. This age-related disparity, coupled with unequal engagement with digital health resources, could contribute to widening gaps in sleep health—reflecting a digital health divide [20]. Therefore, understanding how eHealth literacy influences sleep quality across age groups is critical in addressing this divide.

This study aimed to examine the association between eHealth literacy and sleep quality across age groups among adults aged 18 to 59 years in Shanghai, China. By providing empirical evidence on age-specific associations, this study sought to inform tailored sleep interventions that incorporate eHealth literacy enhancement and address disparities arising from the digital health divide.

## Methods

### Participants and Procedure

This study was conducted between October and December 2022 in Shanghai. Three districts representing urban, periurban, and rural areas were randomly selected. Seven community health service centers from these districts that agreed to participate in the study were included. At each center, community residents were recruited using a convenience sampling approach. Before completing the survey, trained staff provided a detailed explanation of the study’s purpose and requirements, emphasizing the anonymity of responses. Participants were required to sign an informed consent form before proceeding with the questionnaire.

The inclusion criteria were (1) residence in Shanghai, (2) age 18 to 59 years, and (3) provision of informed consent and agreement to participate in the survey. The exclusion criteria were (1) severe hearing or speech impairments and (2) inability to comprehend the survey due to mental or cognitive conditions. Anonymous questionnaires were completed through the online survey platform Wenjuanxing.

The sample size was calculated using the prevalence of poor sleep quality as the primary outcome. On the basis of previous literature, the prevalence of poor sleep quality was estimated to be approximately 35%, with an allowable error of 3.5%. Using the PASS software for cross-sectional survey sample size calculation (NCSS, LLC), the minimum required sample size was determined to be 740. Considering the design effect of 2 due to convenience sampling and an anticipated nonresponse rate of 15%, the adjusted minimum required sample size was 1742.

A total of 1872 eligible participants were invited, and 1810 valid questionnaires were collected, yielding an effective response rate of 96.7%. The final sample size met the minimum requirement for analysis.

### Measurements

#### eHealth Literacy

The Chinese version of the eHealth Literacy Scale (eHEALS), a translation of the original scale developed by Norman and Skinner [21], was used to assess participants’ eHealth literacy [22]. This scale consists of 8 items, each rated on a 5-point Likert scale ranging from 1 (“strongly disagree”) to 5 (“strongly agree”), yielding a total score between 8 and 40. Higher average scores indicate better self-perceived skills, knowledge, and comfort regarding online health information. The eHEALS has good reliability and validity among Chinese adults [23,24]. In this study, the scale showed good internal consistency, with a Cronbach α of 0.98.

#### Sleep Quality

The Pittsburgh Sleep Quality Index (PSQI) was used to measure participants’ sleep quality over the previous month [25]. The scale consists of 19 items evaluating 7 components: subjective sleep quality, sleep latency, sleep duration, habitual sleep efficiency, sleep disturbances, use of sleep medication, and daytime dysfunction. The sum of the component scores yields a total score ranging from 0 to 21, with higher scores indicating poorer sleep quality. According to the recommended cutoff in the original study describing the PSQI, a total score of 5 or lower indicates good sleep quality, whereas a score above 5 indicates poor sleep quality [25]. The PSQI has demonstrated good psychometric robustness and factorial structure among Chinese adults [26,27].

#### Covariates

The selection of covariates was guided by the biopsychosocial theoretical framework [28], which conceptualizes sleep quality as an outcome shaped by biological, psychological, and social determinants. Therefore, covariates were categorized into 3 domains as follows.

##### Biological Factors

Biological factors included sex (male or female), age, and weight status. Weight status was derived from self-reported height and weight, with BMI calculated as weight (kg) divided by height squared (m^2^). Overweight or obesity was defined based on the BMI classification criteria recommended by the Working Group on Obesity in China [29].

##### Psychological Factors

Psychological factors included depressive and anxiety symptoms. Depressive symptoms were assessed using the Patient Health Questionnaire–9, with total scores ranging from 0 to 27. Scores of ≥5, 10, and 15 represent mild, moderate, and severe depressive symptoms, respectively [30]. In this study, the Patient Health Questionnaire–9 demonstrated good internal consistency (Cronbach α=0.964).

Anxiety symptoms were measured using the Generalized Anxiety Disorder–7 scale, with total scores ranging from 0 to 21. Scores of ≥5, 10, and 15 represent mild, moderate, and severe anxiety symptoms, respectively [31]. In this study, the Generalized Anxiety Disorder–7 exhibited good internal consistency (Cronbach α=0.978).

##### Social Factors

Social factors included educational attainment (junior high school or lower, senior high school, or college or higher), employment status (employed or unemployed), family monthly income (<¥5000 [US $707.30], ¥5001-¥9999 [US $707.44-$1414.45], ¥10,000-¥19,999 [US $1414.59-$2829.04], or ≥¥20,000 [US $2829.18]), marital status (either married or single, divorced, or widowed), and residential area (urban, periurban, or rural).

### Statistical Analysis

Participants were first categorized into 3 age groups: emerging adults (18‐29 years) [32], established adults (30‐45 years) [33], and middle-aged adults (46‐59 years). Descriptive statistics were used to summarize their background variables, eHealth literacy, and sleep quality by age group. Given the skewed distribution of eHEALS scores, eHealth literacy was categorized into 3 groups based on IQRs: 25th percentile or below (lowest quartile), 25th to 75th percentile (middle quartiles), and 75th percentile or above (highest quartile). Differences in eHealth literacy and sleep quality among the 3 age groups were examined using chi-square tests.

To examine the association between eHealth literacy and sleep quality, multivariable logistic regression analyses were conducted in a stepwise manner. Model 1 adjusted for biological factors, including sex, age, and BMI. Model 2 incorporated additional adjustments for psychological factors, including depressive and anxiety symptoms. Model 3 further adjusted for social factors, including educational attainment, household monthly income, employment status, marital status, and residential area, to evaluate whether these factors influenced the association between eHealth literacy and sleep quality.

Finally, age-stratified analyses were performed to explore whether the association between eHealth literacy and sleep quality varied across age groups. In sensitivity analyses, we further included potential confounding variables, including chronic disease status and health behaviors (smoking and alcohol consumption), to assess the robustness of the findings.

### Ethical Considerations

The study protocol was approved by the ethics committee of the Xuhui District Center for Disease Control and Prevention (XHLL202205). Written informed consent was obtained from all participants. Participant privacy and confidentiality were strictly protected. All data were anonymized and securely stored, with access limited to the research team.

## Results

### Descriptive Characteristics of the Sample

Table 1 presents the sample characteristics. Of the 1810 participants, 673 (37.2%) were male, and 1137 (62.8%) were female, with a mean age of 40.0 (SD 10.1) years. Of these, 15.7% (285/1810) were emerging adults (18‐29 years), 53.3% (965/1810) were established adults (30-45 years), and 30.9% (560/1810) were middle-aged adults (45‐59 years). Most had a college degree or higher (n=1351, 74.6%), were employed (n=1533, 84.7%), and were married (n=1429, 79%). Approximately half (n=927, 51.2%) reported a monthly household income of ≥¥10,000 (US $1414.59). Regarding residence, 20.7% (375/1810) lived in urban areas, 30.4% (550/1810) lived in periurban areas, and 48.9% (885/1810) lived in rural areas. A total of 21.2% (384/1810) had moderate to severe depressive symptoms, and 15% (271/1810) had moderate to severe anxiety symptoms.

**Table 1.** Sample characteristics by age group (N=1810).

	Total, n (%)	Emerging adults (n=285), n (%)	Established adults (n=965), n (%)	Middle-aged adults (n=560), n (%)
Sex				
Male	673 (37.2)	132 (46.3)	345 (35.8)	196 (35.0)
Female	1137 (62.8)	153 (53.7)	620 (64.2)	364 (65.0)
Weight status				
Normal weight or underweight	1159 (64.0)	191 (67.0)	637 (66.0)	331 (59.1)
Overweight	514 (28.4)	65 (22.8)	254 (26.3)	195 (34.8)
Obesity	137 (7.6)	29 (10.2)	74 (7.7)	34 (6.1)
Depressive symptoms				
None	776 (42.9)	113 (39.6)	399 (41.3)	264 (47.1)
Mild	650 (35.9)	91 (31.9)	349 (36.2)	210 (37.5)
Moderate	135 (7.5)	18 (6.3)	79 (8.2)	38 (6.8)
Severe	249 (13.8)	63 (22.1)	138 (14.3)	48 (8.6)
Anxiety symptoms				
None	971 (53.6)	133 (46.7)	500 (51.8)	338 (60.4)
Mild	568 (31.4)	91 (31.9)	310 (32.1)	167 (29.8)
Moderate	190 (10.5)	43 (15.1)	110 (11.4)	37 (6.6)
Severe	81 (4.5)	18 (6.3)	45 (4.7)	18 (3.2)
Educational attainment				
Junior high school or lower	176 (9.7)	11 (3.9)	39 (4.0)	126 (22.5)
Senior high school	283 (15.6)	17 (6.0)	105 (10.9)	161 (28.8)
College or higher	1351 (74.6)	257 (90.2)	821 (85.1)	273 (48.8)
Employment status				
Employed	1533 (84.7)	248 (87.0)	924 (95.8)	361 (64.5)
Unemployed	277 (15.3)	37 (13.0)	41 (4.2)	199 (35.5)
Family monthly income				
<¥5000 (US $707.30)	349 (19.3)	53 (18.6)	152 (15.8)	144 (25.7)
¥5001-¥9999 (US $707.44-$1414.45)	534 (29.5)	96 (33.7)	285 (29.5)	153 (27.3)
¥10,000-¥19,999 (US $1414.59-$2829.04)	543 (30.0)	85 (29.8)	302 (31.3)	156 (27.9)
≥20,000 (US $2829.18)	384 (21.2)	51 (17.9)	226 (23.4)	107 (19.1)
Marital status				
Married	1429 (79.0)	82 (28.8)	834 (86.4)	513 (91.6)
Single, divorced, or widowed	381 (21.0)	203 (71.2)	131 (13.6)	47 (8.4)
Residential area				
Urban	375 (20.7)	43 (15.1)	197 (20.4)	135 (24.1)
Periurban	550 (30.4)	96 (33.7)	289 (29.9)	165 (29.5)
Rural	885 (48.9)	146 (51.2)	479 (49.6)	260 (46.4)
eHealth literacy score				
Below the 25th percentile	440 (24.3)	79 (27.7)	221 (22.9)	140 (25.0)
Between the 25th and 75th percentiles	909 (50.2)	117 (41.1)	472 (48.9)	320 (57.1)
Above the 75th percentile	461 (25.5)	89 (31.2)	272 (28.2)	100 (17.9)
Sleep quality				
Good	1124 (62.1)	190 (66.7)	617 (63.9)	317 (56.6)
Poor	686 (37.9)	95 (33.3)	348 (36.1)	243 (43.4)

The median score on the eHEALS was 32 (IQR 28-40). The prevalence of poor sleep quality was 37.9% (686/1810). Chi-square analysis revealed significant associations between age group and both eHealth literacy (*χ*^2^_4_=32.0; *P*<.001) and sleep quality (*χ*^2^_2_=11.1; *P*=.004). Compared to younger adults, a lower proportion of middle-aged adults had eHealth literacy scores above the 75th percentile. Additionally, the proportion of middle-aged adults reporting poor sleep quality was higher than that of younger adults.

### Association Between eHealth Literacy and Poor Sleep Quality: Multimodel Regression

The association between eHealth literacy and poor sleep quality was examined using multimodel logistic regression (Table 2). In model 1, after adjusting for biological factors, participants with eHealth literacy scores between the 25th and 75th percentiles (odds ratio [OR] 1.876, 95% CI 1.463-2.406, *P*<.001) and those with scores below the 25th percentile (OR 2.289, 95% CI 1.726-3.037, *P*<.001) had a significantly higher likelihood of reporting poor sleep quality compared to those with scores above the 75th percentile. After further adjusting for psychological factors in model 2, this association remained statistically significant (OR 1.574, 95% CI 1.204-2.058, *P*<.001 for scores between the 25th and 75th percentiles; OR 1.526, 95% CI 1.115-2.088, *P*=.008 for scores below the 25th percentile). The association persisted even after additional adjustment for social factors in model 3 (OR 1.594, 95% CI 1.216-2.089, *P*<.001 for scores between the 25th and 75th percentiles; OR 1.584, 95% CI 1.149-2.182, *P*=.005 for scores below the 25th percentile).

**Table 2.** Association between eHealth literacy and poor sleep quality using multivariable logistic regression (N=1810).

	Model 1^a^		Model 2^b^		Model 3^c^	
	OR^d^ (95% CI)	*P* value	OR (95% CI)	*P* value	OR (95% CI)	*P* value
eHealth literacy						
Below the 25th percentile	Reference	—^e^	Reference	—	Reference	—
Between the 25th and 75th percentiles	1.876 (1.463-2.406)	<.001	1.574 (1.204-2.058)	<.001	1.594 (1.216-2.089)	<.001
Above the 75th percentile	2.289 (1.726-3.037)	<.001	1.526 (1.115-2.088)	.008	1.584 (1.149-2.182)	.005
Sex						
Male	Reference	—	Reference	—	Reference	—
Female	1.050 (0.852-1.294)	.65	1.089 (0.878-1.351)	.44	1.068 (0.856-1.332)	.56
Age	1.012 (1.002-1.022)	.02	1.020 (1.010-1.031)	<.001	1.030 (1.016-1.044)	<.001
Weight status						
Normal weight or underweight	Reference	—	Reference	—	Reference	—
Overweight	0.875 (0.698-1.097)	.25	0.848 (0.671-1.071)	.17	0.852 (0.673-1.079)	.18
Obesity	0.998 (0.684-1.454)	.99	1.061 (0.718-1.567)	.77	1.099 (0.740-1.632)	.64
Depressive symptoms						
None	—	—	Reference	—	Reference	—
Mild	—	—	2.276 (1.699-3.050)	<.001	2.297 (1.711-3.085)	<.001
Moderate	—	—	3.499 (2.199-5.566)	<.001	3.402 (2.132-5.429)	<.001
Severe	—	—	2.082 (1.156-3.749)	.02	2.092 (1.156-3.784)	.02
Anxiety symptoms						
None	—	—	Reference	—	Reference	—
Mild	—	—	1.236 (0.923-1.654)	.16	1.243 (0.927-1.667)	.15
Moderate	—	—	1.620 (0.912-2.876)	.10	1.641 (0.920-2.924)	.09
Severe	—	—	1.632 (0.795-3.348)	.18	1.741 (0.844-3.594)	.13
Educational attainment						
Junior high school or lower	—	—	—	—	Reference	—
Senior high school	—	—	—	—	0.654 (0.432-0.990)	.045
College or higher	—	—	—	—	1.104 (0.742-1.642)	.63
Employment status						
Employed	—	—	—	—	Reference	—
Unemployed	—	—	—	—	1.211 (0.892-1.645)	.22
Family monthly income						
<¥5000 (US $707.30)	—	—	—	—	Reference	—
¥5001-¥9999 (US $707.44-$1414.45)	—	—	—	—	1.010 (0.741-1.375)	.95
¥10,000-19,999 (US $1414.59-$2829.04)	—	—	—	—	1.004 (0.728-1.386)	.98
≥¥20,000 (US $2829.18)	—	—	—	—	1.132 (0.794-1.614)	.49
Marital status						
Married	—	—	—	—	Reference	—
Single, divorced, or widowed	—	—	—	—	1.423 (1.073-1.889)	.01
Residential area						
Urban	—	—	—	—	Reference	—
Periurban	—	—	—	—	0.824 (0.620-1.097)	.19
Rural	—	—	—	—	0.871 (0.664-1.143)	.32

a Adjusting for sex and age.

b Adjusting for sex, age, depressive symptoms, and anxiety symptoms.

c Adjusting for sex, age, depressive symptoms, anxiety symptoms, educational attainment, employment status, family monthly income, marital status, and residential area.

d OR: odds ratio.

e Not applicable.

In the fully adjusted model (model 3), several covariates were also associated with poor sleep quality. Specifically, age (OR 1.030, 95% CI 1.016-1.044), depressive symptoms (OR 2.297, 95% CI 1.711-3.085 for mild; OR 3.402, 95% CI 2.132-5.429 for moderate; OR 2.092, 95% CI 1.156-3.784 for severe), educational attainment (OR 0.654, 95% CI 0.432-0.990 for senior high school), and marital status (OR 1.423, 95% CI 1.073-1.889 for single, divorced, or widowed) were associated with poor sleep quality.

### Age-Stratified Analysis of the Association Between eHealth Literacy and Poor Sleep Quality

Table 3 presents the age-stratified analysis of the association between eHealth literacy and sleep quality across the 3 age groups. Among emerging adults, participants with eHealth literacy scores between the 25th and 75th percentiles (OR 2.491, 95% CI 1.133‐5.479, *P*=.02) and those with scores below the 25th percentile (OR 2.975, 95% CI 1.230‐7.195, *P*=.02) had significantly higher odds of reporting poor sleep quality compared with those with scores above the 75th percentile.

**Table 3.** Association between eHealth literacy and poor sleep quality using multivariable logistic regression stratified by age.

	Emerging adults (n=285)		Established adults (n=965)		Middle-aged adults (n=560)	
	OR^a^ (95% CI)	*P* value	OR (95% CI)	*P* value	OR (95% CI)	*P* value
eHealth literacy						
Above the 75th percentile	Reference	—^b^	Reference	—	Reference	—
Between the 25th and 75th percentiles	2.491 (1.133-5.479)	.02	1.439 (1.001-2.067)	.049	1.651 (0.985-2.770)	.06
Below the 25th percentile	2.975 (1.230-7.195)	.02	1.303 (0.834-2.036)	.24	1.639 (0.901-2.980)	.11
Sex						
Male	Reference	—	Reference	—	Reference	—
Female	0.565 (0.303-1.053)	.07	1.150 (0.844-1.569)	.38	1.198 (0.793-1.810)	.39
Age	1.087 (0.970-1.218)	.15	1.011 (0.979-1.045)	.51	1.021 (0.968-1.080)	.45
Weight status						
Normal weight or underweight	Reference	—	Reference	—	Reference	—
Overweight	1.047 (0.500-2.193)	.90	0.760 (0.541-1.069)	.12	0.924 (0.629-1.360)	.69
Obesity	1.354 (0.482-3.801)	.57	1.040 (0.604-1.789)	.89	1.186 (0.551-2.550)	.66
Depressive symptoms						
None	Reference	—	Reference	—	Reference	—
Mild	4.148 (1.501-11.467)	.006	2.386 (1.570-3.627)	<.001	1.933 (1.202-3.110)	.007
Moderate	6.356 (1.573-25.685)	.009	3.407 (1.796-6.463)	<.001	3.102 (1.324-7.270)	.009
Severe	4.389 (1.019-18.904)	.047	1.594 (0.690-3.681)	.28	2.773 (0.834-9.210)	.10
Anxiety symptoms						
None	Reference	—	Reference	—	Reference	—
Mild	1.361 (0.539-3.438)	.51	1.219 (0.807-1.842)	.35	1.263 (0.774-2.060)	.35
Moderate	1.023 (0.235-4.447)	.98	2.361 (1.058-5.271)	.04	0.996 (0.323-3.070)	.99
Severe	3.747 (0.674-20.832)	.13	2.408 (0.874-6.637)	.09	0.567 (0.122-2.630)	.47
Educational attainment						
Junior high school or lower	Reference	—	Reference	—	Reference	—
Senior high school	0.095 (0.007-1.282)	.08	0.730 (0.307-1.736)	.48	0.696 (0.420-1.150)	.16
College or higher	1.277 (0.279-5.848)	.75	1.580 (0.715-3.491)	.26	0.801 (0.468-1.370)	.42
Employment status						
Employed	Reference	—	Reference	—	Reference	—
Unemployed	1.605 (0.600-4.294)	.35	1.152 (0.578-2.299)	.69	1.081 (0.679-1.720)	.74
Family monthly income						
<¥5000 (US $707.30)	Reference	—	Reference	—	Reference	—
¥5001-¥9999 (US $707.44-$1414.45)	0.557 (0.240-1.289)	.17	0.997 (0.627-1.584)	.99	1.101 (0.660-1.840)	.71
¥10,000-¥19,999 (US $1414.59-$2829.04)	0.530 (0.216-1.299)	.17	0.904 (0.560-1.460)	.68	1.296 (0.762-2.200)	.34
≥¥20,000 (US $2829.18)	0.729 (0.266-1.998)	.54	1.100 (0.657-1.841)	.72	1.336 (0.729-2.450)	.35
Marital status						
Married	Reference	—	Reference	—	Reference	—
Single, divorced, or widowed	0.964 (0.469-1.978)	.92	1.736 (1.150-2.621)	.009	1.354 (0.711-2.580)	.36
Residential area						
Urban	Reference	—	Reference	—	Reference	—
Periurban	0.682 (0.294-1.584)	.37	0.816 (0.546-1.219)	.32	0.856 (0.527-1.390)	.53
Rural	0.547 (0.242-1.235)	.15	0.868 (0.588-1.281)	.48	0.897 (0.564-1.430)	.65

a OR: odds ratio.

b Not applicable.

Among established adults, participants with scores below the 25th percentile showed no statistically significant association (OR 1.303, 95% CI 0.834‐2.036, *P*=.24), whereas the group between the 25th and 75th percentiles showed a positive association (OR 1.439, 95% CI 1.001‐2.067, *P*=.049). However, this association was not statistically significant among middle-aged adults (OR 1.651, 95% CI 0.985‐2.770, *P*=.06 for scores between the 25th and 75th percentiles; OR 1.639, 95% CI 0.901‐2.980, *P*=.11 for scores below the 25th percentile).

### Sensitivity Analysis

Sensitivity analyses adjusting additionally for smoking, alcohol consumption, and chronic disease status are presented in Multimedia Appendix 1. Among emerging adults, lower eHealth literacy remained significantly associated with higher odds of poor sleep quality (OR 2.330, 95% CI 1.045‐5.197, *P*=.04 for scores between the 25th and 75th percentiles; OR 2.564, 95% CI 1.017‐6.464, *P*=.046 for scores below the 25th percentile). Among established adults, lower eHealth literacy did not show a statistically significant association after additional adjustment (OR 1.377, 95% CI 0.953‐1.991, *P*=.09 for scores between the 25th and 75th percentiles; OR 0.776, 95% CI 0.776‐1.930, *P*=.39 for scores below the 25th percentile). Among middle-aged adults, results also remained nonsignificant (OR 1.539, 95% CI 0.910‐2.600, *P*=.11 for scores between the 25th and 75th percentiles; OR 1.476, 95% CI 0.802‐2.710, *P*=.21 for scores below the 25th percentile).

## Discussion

### Principal Findings

This study investigated the association between eHealth literacy and sleep quality among adults aged 18 to 59 years in Shanghai, China. Overall, lower eHealth literacy scores were associated with a higher likelihood of poor sleep quality even after adjusting for biological, psychological, and social factors. The stratified analysis revealed that this association was significant among younger adults but not among middle-aged adults. These findings provide empirical evidence supporting the role of eHealth literacy as a potential determinant of sleep quality, particularly among younger populations.

The significant association observed in this study is consistent with prior research linking limited health literacy to poorer sleep outcomes and increased sleep disturbances [34,35]. While existing studies have largely focused on traditional health literacy, emerging research suggests that eHealth literacy may play a comparable role in health management in digital contexts [36]. Extending previous findings that link eHealth literacy to better adherence to sleep hygiene practices [13], our results suggest a more direct association between eHealth literacy and overall sleep quality. Individuals with higher eHealth literacy are better equipped to critically evaluate online health information and adopt evidence-based sleep practices. In contrast, limited eHealth literacy may increase vulnerability to online misinformation and suboptimal sleep practices, ultimately leading to poorer sleep outcomes.

Beyond eHealth literacy, several other factors, including age, educational level, marital status, and depressive symptoms, were also associated with sleep quality in the overall model, consistent with findings from previous research [37,38]. Among these factors, depressive symptoms emerged as a well-established and particularly strong predictor of sleep disturbances [39]. Individuals with mild to severe depressive symptoms had approximately 2 to 3 times higher odds of reporting poor sleep quality compared with those without depressive symptoms. This strong psychological effect may have attenuated the independent contributions of other covariates when adjusting simultaneously. In addition, prior studies have shown that individuals with lower eHealth literacy tend to experience greater psychological distress [40], partly due to the misuse of misleading or low-quality information encountered online. These patterns suggest that mental health may play an important role in the pathway through which eHealth literacy relates to sleep quality.

In the age-stratified analysis, lower eHealth literacy was associated with poorer sleep quality only among emerging and established adults. This finding aligns with those of prior research indicating that younger adults typically engage more actively with digital health information [15,16] and rely more on online resources for health-related decisions. In contrast, middle-aged and older adults tend to depend more on traditional health care resources [41], making their sleep quality less influenced by online health information use. Furthermore, this age-specific association may also reflect distinct underlying mechanisms of sleep disturbances. Among middle-aged adults, sleep disturbance is more frequently attributed to age-related neurophysiological and neurochemical changes (eg, reduced sleep duration and increased fragmentation) [42]. Such physiologically driven sleep disturbances are only minimally related to eHealth literacy. Conversely, younger adults often experience irregular sleep patterns driven by external demands (eg, academic or occupational stress) [18,43], which may be more amenable to modification through improved eHealth literacy.

The age-specific association between eHealth literacy and sleep quality aligns with broader concerns about the digital health divide [20]. Although digital health technologies offer scalable and cost-effective solutions for health management, their benefits are not equitably distributed across age groups. Structural barriers such as limited access, lower digital confidence, and affordability disproportionately affect marginalized and older populations [44]. As sleep disturbances tend to increase with age, middle-aged adults may face a dual challenge: increased physiological susceptibility to poor sleep and reduced capacity to engage with digital resources. However, existing eHealth interventions aimed at improving sleep outcomes have predominantly targeted younger populations [45-47]. Without targeted support, the expansion of digital health tools may unintentionally widen existing age-related disparities in sleep health.

Our findings highlight the importance of improving eHealth literacy to promote better sleep outcomes. For example, a 6-week online intervention during the COVID-19 pandemic integrated health education and digital skill training to improve university students’ eHealth literacy and related health behaviors [48]. Although short-term sleep improvements were limited, the study highlighted the potential of eHealth literacy–based interventions and the importance of long-term evaluation [48]. Given the age-stratified association observed in our study, tailoring interventions to address age-specific barriers is essential. Middle-aged adults, who have lower digital engagement, may require additional support to effectively benefit from digital tools—such as affordable internet access, community-based digital skill training, and user-friendly interface design [49]. By accounting for the unique needs of different age groups, eHealth literacy can be leveraged to improve health outcomes for all, ultimately advancing digital health equity.

### Strengths and Limitations

This study has several strengths, including adjustment for multiple confounders at different levels and an age-stratified analysis, offering a more nuanced understanding of the association between eHealth literacy and sleep quality. However, several limitations should be acknowledged. First, the cross-sectional design precludes causal inferences between eHealth literacy and sleep quality. Future studies should use longitudinal or experimental designs to clarify temporal relationships and causal pathways between eHealth literacy and sleep outcomes. Second, the study was conducted exclusively among adults in Shanghai, limiting generalizability to other regions with different levels of health literacy. Third, reliance on self-reported measures for eHealth literacy and sleep quality may introduce recall and social desirability biases, potentially affecting the accuracy of the results. Fourth, due to the need to minimize respondent burden, alcohol use and smoking were assessed using frequency-based measures rather than consumption volume. This may not fully capture the potentially nonlinear associations between these behaviors and sleep quality. Fifth, the overall sample size was relatively limited, particularly within certain age groups, which may have affected the statistical precision of the findings. Finally, we did not include key determinants of sleep such as work or study pressure, exercise habits, and online behaviors, which may introduce residual confounding. Future studies should incorporate these psychosocial and behavioral factors to more fully disentangle the association between eHealth literacy and sleep quality.

### Conclusions

This study examined the association between eHealth literacy and sleep quality among adults aged 18 to 59 years in Shanghai, China. Findings showed that lower eHealth literacy was significantly associated with a higher likelihood of reporting poor sleep quality. Age-stratified analysis further revealed that this relationship was significant among younger adults but not among middle-aged adults. These findings underscore the potential of enhancing eHealth literacy as an effective strategy for improving sleep health, particularly when tailored to age-specific needs and digital access levels. Targeted measures to reduce the digital health divide will be essential in promoting more equitable health outcomes across age groups.

## Supplementary material

10.2196/75813 Multimedia Appendix 1 Supplementary table presenting the results of the sensitivity analyses.

## Abbreviations

eHEALS: eHealth Literacy Scale
OR: odds ratio
PSQI: Pittsburgh Sleep Quality Index
