# Supplementary material for: Age-Specific Associations Between eHealth Literacy and Sleep Quality Among Adults: Cross-Sectional Study
Source: J Med Internet Res. 2025 Dec 24;27:e75813. doi: 10.2196/75813 (PMC12735630; doi:10.2196/75813)
Supplement: Multimedia Appendix 1 [file jmir-v27-e75813-s001.docx]

**Supplementary Table 1 Sensitivity analysis**

|  | Emerging adults (n = 285) | | Established adults (n = 965) | | Middle-aged adults (n = 560) | |
| --- | --- | --- | --- | --- | --- | --- |
|  | OR (95% CI) | P value | OR (95% CI) | P value | OR (95% CI) | P value |
| **E-health literacy** |  |  |  |  |  |  |
| >P_75_ | Ref | | Ref | | Ref | |
| P_25_-P_75_ | 2.330 (1.045-5.197) | .04 | 1.377 (0.953-1.991) | .09 | 1.539 (0.910-2.600) | .11 |
| <P_25_ | 2.564 (1.017-6.464) | .046 | 1.224 (0.776-1.930) | .39 | 1.476 (0.802-2.710) | .21 |
| **Sex** |  |  |  |  |  |  |
| Male | Ref | | Ref | | Ref | |
| Female | 0.758 (0.379-1.515) | .43 | 1.586 (1.112-2.261) | .01 | 1.539 (0.941-2.520) | .09 |
| **Age (year)** | 1.067 (0.948-1.201) | .28 | 1.004 (0.970-1.038) | .84 | 1.006 (0.952-1.060) | .85 |
| **Education attainment** |  |  |  |  |  |  |
| ≤ Junior high school | Ref | | Ref | | Ref | |
| Senior high school | 0.029 (0.001-0.603) | .02 | 0.879 (0.357-2.162) | .78 | 0.685 (0.410-1.140) | .15 |
| ≥ College | 1.320 (0.278-6.267) | .73 | 1.990 (0.868-4.561) | .10 | 0.772 (0.446-1.340) | .36 |
| **Employment** |  |  |  |  |  |  |
| Employed | Ref | | Ref | | Ref | |
| Unemployed | 1.428 (0.517-3.944) | .49 | 1.176 (0.581-2.384) | .65 | 1.031 (0.641-1.660) | .90 |
| **Family monthly income** |  |  |  |  |  |  |
| <5000 | Ref | | Ref | | Ref | |
| 5001-9999 | 0.625 (0.261-1.496) | .29 | 0.997 (0.623-1.595) | .99 | 1.047 (0.620-1.770) | .86 |
| 10000-19999 | 0.643 (0.249-1.655) | .36 | 0.855 (0.524-1.394) | .53 | 1.189 (0.689-2.050) | .53 |
| >=20000 | 0.890 (0.308-2.574) | .83 | 1.070 (0.632-1.813) | .80 | 1.198 (0.644-2.230) | .57 |
| **Marital status** |  |  |  |  |  |  |
| Married | Ref | | Ref | | Ref | |
| Single/Divorced/widowed | 0.960 (0.455-2.027) | .91 | 1.745 (1.147-2.656) | .009 | 1.332 (0.697-2.540) | .39 |
| **Residential area** |  |  |  |  |  |  |
| Urban | Ref | | Ref | | Ref | |
| Peri-urban | 0.749 (0.310-1.806) | .52 | 0.816 (0.541-1.231) | .33 | 0.904 (0.551-1.480) | .69 |
| Rural | 0.515 (0.220-1.204) | .13 | 0.901 (0.604-1.345) | .61 | 0.891 (0.557-1.430) | .63 |
| **BMI (kg/m^2^)** |  |  |  |  |  |  |
| Normal/underweight | Ref | | Ref | | Ref | |
| Overweight | 1.116 (0.517-2.409) | .78 | 0.716 (0.504-1.015) | .06 | 0.843 (0.568-1.250) | .40 |
| Obesity | 1.420 (0.473-4.268) | .53 | 0.855 (0.489-1.493) | .58 | 0.945 (0.426-2.100) | .89 |
| **Depressive symptoms** |  |  |  |  |  |  |
| Non | Ref | | Ref | | Ref | |
| Mild | 3.609 (1.242-10.480) | .02 | 2.447 (1.596-3.751) | <.001 | 1.895 (1.169-3.070) | .01 |
| Moderate | 6.994 (1.648-29.685) | .008 | 3.449 (1.800-6.610) | <.001 | 3.017 (1.260-7.220) | .01 |
| Severe | 4.129 (0.918-18.580) | .07 | 1.661 (0.710-3.886) | .24 | 2.668 (0.797-8.930) | .11 |
| **Anxiety symptoms** |  |  |  |  |  |  |
| Non | Ref | | Ref | | Ref | |
| Mild | 1.555 (0.587-4.116) | .37 | 1.193 (0.783-1.818) | .41 | 1.218 (0.739-2.010) | .44 |
| Moderate | 0.910 (0.200-4.144) | .90 | 2.074 (0.913-4.712) | .08 | 0.979 (0.314-3.050) | .97 |
| Severe | 3.825 (0.677-21.617) | .13 | 2.325 (0.830-6.515) | .11 | 0.535 (0.113-2.540) | .43 |
| With chronic disease |  |  |  |  |  |  |
| No | Ref | | Ref | | Ref | |
| Yes | 8.451 (1.927-37.061) | .005 | 1.941 (1.315-2.866) | <.001 | 1.762 (1.198-2.590) | .004 |
| Smoking |  |  |  |  |  |  |
| No | Ref | | Ref | | Ref | |
| Yes | 1.559 (0.599-4.057) | .36 | 1.368 (0.841-2.226) | .21 | 0.781 (0.436-1.400) | .41 |
| Drinking |  |  |  |  |  |  |
| No | Ref | | Ref | | Ref | |
| Yes | 1.620 (0.703-3.732) | .26 | 1.960 (1.329-2.892) | <.001 | 1.972 (1.158-3.360) | .01 |

Abbreviations: OR, odds ratio; CI, confidence interval.
